# Major and Trace Element Content of *Tribulus terrestris* L. Wildlife Plants

**DOI:** 10.3390/plants9121764

**Published:** 2020-12-13

**Authors:** Kirill Tkachenko, Marina Frontasyeva, Atanas Vasilev, Latchezar Avramov, Lei Shi

**Affiliations:** 1 Komarov Botanical Institute of Russian Academy of Sciences, Peter the Great Botanical Gardens, 197376 Saint-Petersburg, Russia; kigatka@gmail.com; 2 Joint Institute for Nuclear Research, Frank Laboratory of Neutron Physics, 141980 Dubna, Russia; 3 Faculty of Physics, Sofia University “St. Kliment Ohridski”, 1164 Sofia, Bulgaria; phys_vasilev@abv.bg; 4 Academician Emil Djakov Institute of Electronics of Bulgarian Academy of Sciences, 1784 Sofia, Bulgaria; avramov@ie.bas.bg; 5 Institute of Botany of Chinese Academy of Sciences, Key laboratory for Plant Resources Botanical Garden, Beijing 100093, China; shilei@ibcas.ac.cn

**Keywords:** herbal medicinal products, epithermal neutron activation analysis, elemental content, bioavailability, complementary foods

## Abstract

The genus *Tribulus* L. (Zygophyllaceae) includes 12 species, the most important of which is *Tribulus terrestris* L. This annual herb grows in temperate and tropical climates, and has a rich chemical composition of biologically active substances and chemical elements. Medicinal plants, and the phytopreparations obtained from them, are becoming more and more popular in world practice as they are used to successfully treat human diseases. Their therapeutic effect is due to the presence in them, of a variety of natural compounds and biologically important trace elements, especially in higher concentrations present in higher doses. *T. terrestris* is becoming more and more popular for the treatment of diseases of the human genital area and sexual dysfunctions. The elemental content in the tissues of leaf, flower, and fruit of *T. terrestris* was determined by using multi-element instrumental epithermal neutron activation analysis. For the first time, 26 essential and trace elements were observed in the plant species collected in Russia (from cultivated) and China (wild growing). It was confirmed that the elemental composition of *T. terrestris* grass varies depending on the habitat (geographic zones). The place of growth affects the accumulation of elements by the plant.

## 1. Introduction

The medicinal value of plants lies in some chemical substances that produce a definite physiological action in the human body. Medicinal plants contain both organic and inorganic constituents. The pharmacological properties of medicinal plants have been attributed to the presence of active chemical constituents that are responsible for important physiological functions in living organisms. The steroidal saponins of *Tribulus terrestris* L. (Zygophyllaceae) are considered the factor responsible for the biological activity of products derived from this plant. Its various parts contain a variety of medicinally important chemical constituents, such as flavonoids, flavonol glycosides, steroidal saponins, and alkaloids. The activity depends on the concentration and the composition of active saponins, which in turn is influenced by the geographical origin of plant material [1,2,3,4,5,6]. Since the levels of Na, Mg, K, Ca, and Zn in analyzed samples of *T. terrestris* are higher than the levels considered as reference, the importance of the contribution by *T. terrestris* in the daily human needs can be considered insignificant. The element determination can be important in controlling the quality of the raw material for standardized extracts (especially heavy metal content) [7,8,9,10,11,12,13].

The Ca, Fe, Zn content of the tested raw materials of *T. terrestris*, although high, cannot be used to supplement the intake, since any plant sources that have a high content of bi- or tri-valent ions have a low bioavailability due to their content of phytic acid, polyphenols, and tannins, which drastically limits absorption. Despite other favorable effects, phytic acid is an antinutrient regarding mineral intake [10,12,13].

A number of herbal preparations from *T. terrestris* have been produced in Bulgaria for many years. Consequently, many works have been devoted to the study of this species in nature [14,15]. It has been shown that this plant species accumulates flavonoids, which provides biological activity [16].

Trace element concentrations present in medicinal plants are of great importance for understanding their pharmacological actions [17,18]. The chemical constituents in plants, including metal ions, are particularly responsible for medical and nutritional properties and for toxicity as well. The metals also play an important role in the plants themselves. The heavy metals can directly impair mental and neurological function, by influencing neurotransmitter production and by utilizing and altering numerous metabolic body processes. The systems in which toxic metal elements can induce impairment and dysfunction includes the blood and cardiovascular detoxification pathways (colon, liver, kidney, and skin), endocrine energy production pathways, and the enzymatic, gastrointestinal, immune, nervous, reproductive, and urinary systems.

Trace elements play a very important role in the formation of the active chemical constituents present in medicinal plants. The quantitative estimation of various trace element concentrations is important for determining the effectiveness of the plants in treating various diseases, and understanding their pharmacological action. Raw materials from medicinal plants are used in the preparation of various modern drugs and they have even been used as the principal source of raw materials for conventional drugs in the developing countries where these plants are easily found in local markets. They are available in the mixture of medicinal plant extracts and in capsule form; therefore, they are easy to use and the industry promises more quick effects. There is great interest in tracing the essential elements and their composition in medicinal science; it is believed that the great majority of these elements are key components of an essential enzyme system or vital bio-chemical function.

**Figure 1:**
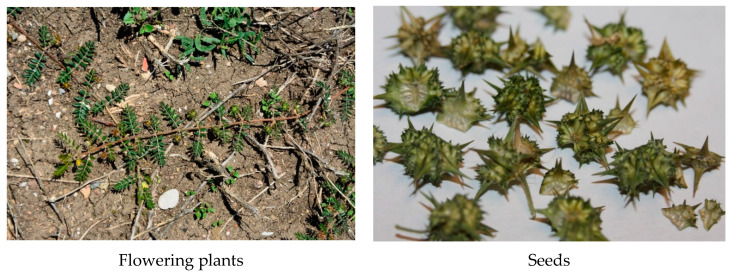
*Tribulus terrestris* L.

*Tribulus terrestris* L. (family Zygophyllaceae) (Figure 1) is a widespread pantropic weed, which grows as a weed on sandy soils in subtropical, and desert climate regions around the world: in South and East Europe (Mediterranean), in Asia (India, China, Vietnam etc.), South Africa, USA, Mexico. *T. terrestris* L. is widely known as a medicinal plant whose herb is the basis for a number of effective preparations based on steroidal glycosides and steroidal saponins [9,19,20,21,22,23,24,25,26,27,28,29,30,31,32].

In Siddha medicine, *T. terrestris* is known in Tamil as *Nerunjil, yanai vanangi, thirikandam, siru nerunjil*. In India—as Gokshur or Gokharu (in Sanskrit—gokshura, means the “cow’s hoof”, possibly because the small thorns tend to be stuck on grazing animals). This whole plant is used in the form of decoction to treat urinary tract infections, urolithiasis, dysmenorrhea, and edema. In Ayurvedic pharmacology, *T. terrestris* is used as a powder form of the aerial parts, particularly the fruits. In Europe, this species also named as puncture vine, “plant viagra” [31].

*T. terrestris* is a traditional Asiatic medicinal plant, commonly used for skin disease, gonorrhea, heart disease, liver, and urinary tract infection [30], treating eye trouble, edema, skin itch, high blood pressure, and cardiovascular diseases [33], fruit—as a diuretic, sexual dysfunction, and veiling [34]. In traditional Chinese medicine, the fruit is used to treat the liver, invigorate the blood, dispel wind, brighten the eyes, and against sexual impotence, edemas, abdominal distention, cardiovascular diseases and to relieve itching [4]. The commonly available weed, *T. terrestris,* is of significant value in many traditional systems of medicine (Ayurveda, Traditional Chinese Medicine, Siddha, and Unani), where it is used as a diuretic, aphrodisiac, antiurolithic, immunomodulatory, antihypertensive, antihyperlipidemic, antidiabetic, hepatoprotective, anticancer, anthelmintic, antibacterial, analgesic, and anti-inflammatory.

Elemental analyses of herbals are of therapeutic importance. The aim of this investigation is to determine the elemental concentration of medicinally important herbals tissues of *T. terrestris* L. using one of most advanced analytical technique, epithermal neutron activation analysis [35].

## 2. Materials and Methods

### 2.1. Sampling and Sample Preparation

Herbs of *Tribulus terrestris* L. (Zygophyllaceae) were collected in 2015 from the wild in various regions of China. The first two samples were collected in the province of Inner Mongolia, in the vicinity of the city of Bao Tou, where gray-brown desert soils, gray soils, chestnut, light meadow, mountain-steppe, and mountain-meadow soils dominate. Sample no. 1 was collected in June and sample no. 2 was collected in July.

Sample no. 3 was collected in August in the vicinity of Beijing, in the Xian Shan Mountains region (near the Botanical Garden of the Institute of Botany of the Academy of Sciences of China), where the soils are highly acidic and depleted in nutrients, such as potassium, phosphorus, and nitrogen. The humus content in the upper horizon reaches 6–9%. Due to the fact that red soils are often located on the slopes of mountains and foothills, they are prone to erosion, which sharply reduces their fertility. All of the plant material samples were collected away from possible sources of industrial pollution. Sample no. 4 (also collected in August) was of plants grown from seeds in the Peter the Great Botanical Garden of the Botanical Institute of RAS (the Russian Academy of Sciences) (St. Petersburg, Russia). The plants were grown on an artificial soil mixture of sod, leafy soil, sand with the addition of peat. Seeds for the cultivation of *T. terrestris* were brought from China in 2014 and the plants were grown in the collection nursery of Medicinal plants of the Peter the Great Botanical Garden in St. Petersburg, Russia, and the herbs collected in August 2015. The plant material (branches, flowers, and leaves in equal amount) was not washed. Plants were air-dried in a clean drying chamber and then oven dried at 40 °C for 48 h until constant weight. The dried samples were ground into fine powder in an agate ball mill. Two sub-samples of each plant were pressed into thick pellets using a press-form of 13 mm diameter.

Vegetation samples for short-term irradiation were heat-sealed in polyethylene foil bags and the samples for long-term irradiation were packed in aluminum cups.

### 2.2. Epithermal Neutron Activation Analysis (ENAA)

The analytical procedures and the basic characteristics of the pneumatic system employed at the pulsed fast reactor IBR-2M are described in detail elsewhere [35]. Two types of irradiation were carried out. One is a short irradiation for 3–5 min to determine short-lived isotopes (Al, Ca, Cl, I, Mg, Mn, and V). After a decay-period of 5–7 min the irradiated samples were measured twice, first for 3–5 min and then for 10–15 min. A long-irradiation of 4–5 days was used to analyze for long-lived radionuclides. After irradiation the samples were re-packed and measured twice: first after 4–5 days for 40–50 min to determine As, Br, K, La, Na, Mo, Sm, U, and W and after 20 days for 2.5–3 h to determine Ba, Ce, Co, Cr, Cs, Fe, Hf, Ni, Rb, Sb, Sc, Sr, Ta, Tb, Th, Yb, and Zn. The gamma spectra of induced activity were measured with an HPGe detector with a resolution of 1.9 keV for the ^60^Co 1332 keV line.

The processing of spectra data and calculation of elemental concentrations were performed using software developed in FLNP, JINR [36]. Certified reference materials and flux comparators were used to determine the concentrations of elements by relative method of calculations.

### 2.3. Quality Control of ENAA

In order to evaluate the precision and accuracy of the results, the certified reference materials and standards were used, namely NIST SRM 1575a—Trace Elements in Pine Needles, NIST SRM 1547—Peach Leaves, NIST SRM 1633b—Constituent Elements in Coal Fly Ash, NIST SRM 1632c—Trace Elements in Coal (Bituminous), IRMM SRM 667—Estuarine Sediment, NIST SRM 2711—Montana Soil, NIST SRM 2710—Montana Soil. Table 1 shows the differences between certified and calculated values of concentrations, where “SRM” were used as standards for calculations of concentrations for SRMs in the column “Sample”. Most differences between certified and obtained values are lower than 2 σ, but for Sc, Sm, and U the relative differences are 20–27%. There are no such data for elements Mo and Cd because their certified values are in the irradiated SRM only.

## 3. Results

Table 2 contains the results of ENAA of the *T. terrestris* herbs. The concentrations of the main elements in the wild *T. terrestris* herbs are given in mg/kg, depending on the harvesting site, whereas the concentration of toxic elements are presented in mg/kg//σ, % according to the organs of *T. terrestris*, depending on the site of collection.

Samples in column no. 1 were collected in the province of Inner Mongolia, in the vicinity of the city of Bao Tou (was collected in June); no. 2, collected in July; no. 3, collected in the vicinity of Beijing, in the Xian Shan Mountains region (in August). No. 4 were collected in the Peter the Great Botanical Garden Botanical Institute of RAS (St. Petersburg, Russia) (in August).

From the data in Table 2 it can be seen that in natural samples (from China) the content of elements such as Na, Mg, Cl, K, Ca, and Fe is higher than in plants grown in the Botanical Garden (Russia). However, the content of magnesium and zinc is higher in the Russian samples, 2–3 times higher than in the Chinese ones.

It also follows from the data in Table 2 that the toxic elements in the sample from Russia are many times lower than in those taken from China. However, the As content was found to be higher. The content of Ba and Th is lower than 10 times, Sr—six times, V—five times.

## 4. Discussion

It was shown earlier that the content of such macronutrients as K—41.5 (mg/g), Ca—36.7, Mg—6.1, Fe—0.3 were determined in the *T. terrestris* herbs [38]. Moreover, the concentrations of the trace elements (the coefficient of accumulation of microelements) are as follows: Mn—0.11, Cu—0.52, Zn—2.49, Co—0.09, Mo—0.90, Cr—0.48, Al—0.20, Ba—11.83, V—0.01, Se—5.50, Ni—0.51, Sr—6.17, Cd—8.00. It is indicated that they accumulate Zn, Cd, Sr, Ba, Se (especially Ba and Sr). A comparison of the data obtained with the published data [39] shows that the studied samples of *T. terrestris* from China accumulate the same substances in comparable amounts.

From the data in Table 2, it can be seen that the level of Na, K, Fe decreases in the grass on the gradient of summer months in the *T. terrestris* grass collected in China. The grown plants of *T. terrestris* in the conditions of St. Petersburg accumulate much fewer basic elements, in comparison with wild plants.

The results obtained were compared to the “Reference Plant” data [37]. This comparison shows that, in all studied plant concentrations of elements, such as Al, Cl, Br, Mo, and Se exceeds the “Reference plant” values. Each essential element and its concentration in the studied plants and its estimated intake by each type of consumer is discussed in following sections.

- Na: sodium is an important element for the maintenance of acid-base equilibrium and of osmotic pressure of body liquids [40].
- Mg: magnesium is known to catalyze important reactions related to muscle contraction and its deficiency in human metabolism can cause neuromuscular dysfunctions [41].
- Al: aluminum toxicity is a major constraint for crop production in acidic soil worldwide. When the soil pH is lower than 5, Al^3+^ is released to the soil and enters into root tip cell ceases root development of plant. In acid soil with high mineral content, Al is the major cause of phytotoxicity.
- Cl: chlorine (Cl) occurs predominantly as Cl^-^ in soil and plant. At adequate levels of supply, Cl improves the yields and quality of many crops, such as onions and cotton if the soils are deficient in this nutrient. When excessive, Cl can be as a major component of salinity stress and toxic to plants.
- K: potassium is one of the most abundant elements in the plant materials. The high concentration of potassium in plants is needed for many essential processes, including enzyme activation, photosynthesis, water use efficiency, starch formation, and protein synthesis. Moreover, K is responsible for regulating osmotic pressure of body fluids, and for maintaining cardiac rhythm, and in constipation. The recommended daily allowance is 550–5625 mg [42], but analyzed samples contain from 12,100 to 26,400 mg/kg potassium.
- Ca: in the human metabolism, Ca is known to be an important constituent of bones and teeth, to be participated in the biochemical blood clotting process and to be responsible for proper nerve and muscle function [43]. It is also necessary for the absorption of dietary vitamin B. The recommended dietary allowance (RDA) is 800–1200 mg for adult [42].
- Sc: the biological role of scandium is poorly known. Under ordinary conditions, the concentration of Sc in plants is very low. It was shown that an increase of Sc in soil may result in both a significant increase of Sc content in the plants and variations in concentrations of essential nutrients in all parts of plants.
- V: vanadium, although essential for growth and chlorophyll formation in unicellular green algae, reveals toxic influences on cell division of *Chlorella pyrenoidosa*, these disturbances arising in the same range of V-concentrations as the known positive effects of the trace metal.
- Mn: manganese is an essential element required for various biochemical processes. It helps in eliminating fatigue and reduces nervous irritability [44]. The requirement of Mn for an adult is 1.0–5.0 mg [42].
- Fe: the role of iron in the body and is an essential component of hemoglobin. It facilitates the oxidation of carbohydrates, protein, and fat to control body weight, which is a very important factor in diabetes [45]. Fe is important because it eliminates phlegm and strengthens the function of stomach. Hence, the daily intake of iron is necessary. The RDA is 10–18 mg [42].
- Co: cobalt acts as a preprophase poison and thus retards the process of karyokinesis and cytokinesis. The action of cobalt on plant cells is mainly turbagenic. Cobalt compounds act on the mitotic spindle, leading to the formation of chromatin bridges, fragmentation, and sticky bridges at anaphase and binucleate cells.
- Zn: zinc is essential to all organisms and it is an important trace element having a definite role in the metabolism, growth, and development. It is an essential component of over 200 enzymes having both catalytically and structural roles. Zn deficiency is characterized by recurrent infections, lack of immunity, and poor growth. The RDA for Zn is 15 mg [42].
- Se: selenium is a necessary component for healthy reproductive status and it aids in normalizing both testosterone and estrogen levels. Selenium aids in the removal of lipids and phospholipids (vitamin C). It is also involved in the metabolism of glucose, collagen, folic acid, and certain amino acids. Finally, selenium has been proven a powerful anti-carcinogenic supplement inhibiting cancer cells and reducing the risk of several cancers including prostate, breast, and uterine cancers. The requirement of Se for an adult is 0.02–0.2 mg/day [42]. The selenium concentration in the plant samples analyzed ranges from 0.08 to 0.12 mg/kg.
- Rb: rubidium has no known biological role but has a slight stimulatory effect on metabolism, probably because it is like potassium. The two elements are found together in minerals and soils, although potassium is much more abundant than rubidium. Plant will adsorb rubidium quite quickly. When stresses by deficiency of potassium some plants, such as sugar beet, will respond to the addition of rubidium.
- Sr: strontium (Sr) is not an essential element to plants; however, high soil-to-crop transfer factors (TFs, concentration ratios between crop and soil) of this element are often found because calcium, in the same element group as Sr, is an essential element for plants.
- Mo: molybdenum is essential for most organisms and occurs in more than 60 enzymes catalyzing diverse oxidation-reduction reactions. Mo is also a component of coenzyme that is essential for the activity of xanthine oxidase, sulfite oxidase and aldehyde oxidase [46,47]. It acts as a detoxification agent in the liver as a part of the sulfite oxidase enzyme and it possibly retards degenerative diseases, cancer, and aging.
- Cs: cesium is reported to be easily absorbed by plants and algal cells via potassium transport systems as an analogue of potassium.
- Ba: because it forms insoluble salts with other common components of the environment, such as carbonate and sulfate, barium is not mobile and poses little risk.
- REEs: rare earth elements (REEs) content is one of the main factors that affect four kinds of functions of herbal medicine [48]. The optimum concentration of some REEs can promote the growth of medicinal plants efficiently. As can be seen from the analytical results, there are differences in content for different REEs in plant samples. Most of the concentration of REEs in studied plants are below the maximum permissible levels (MPL), but content of Eu in all samples higher than value in “Reference value”.
- Ta: only tiny amounts of tantalum are taken by plants: the amount in vegetation rarely exceeds 5 ppb.

Our results are consistent with those of Affaf et al., 2019 [49]. It has been established that potassium and calcium accumulate in the highest amounts among the macroelements in *T. terrestris* grass samples; they account for about 90% of the total content of elements in the plant. It was revealed that the distribution of macro and microelements in a plant differs significantly depending on the place, time, and growing conditions.

The role of selenium, both in plant life and in the treatment of human diseases, is widely studied [50,51]. The high selenium content in the raw material probably determines the antioxidant effect. Moreover, consumption of the herb is possible with thyroid disease [52,53,54,55,56,57,58].

## 5. Conclusions

In the raw materials of the *T. terrestris,* the maximum accumulation of macro- and microelements was observed in June. By August, the content of Na, Mg, K, Cl, and Ca decreases. Wild plants *T. terrestris* (natural growth in China) accumulate much more basic elements. The conditions of culture in the Russia (Botanical garden in North-West of Russia, St. Petersburg) do not contribute to the accumulation of basic elements in herb of the *T. terrestris.*

The study of the elemental composition of the creeping grass, *T. terrestris,* showed that the habitats (geographical zones), in which the studied samples of raw materials were collected, affect the accumulation of elements by the plant. This confirmed the position given in other works [9,49].

The high Se content may explain the effectiveness of herbal preparations from *T. terrestris* for the prevention and treatment of sexual dysfunctions.

The content of Mg and Zn is higher in the Russian samples, 2–3 times higher than in the Chinese ones.

The toxic elements at the raw material in the sample from Russia are many times lower than in those taken from China. However, the As content was found to be higher. The content of Ba and Th is lower than 10 times, Sr—six times, V—five times.

## Figures and Tables

**Table 1:** Epithermal Neutron Activation Analysis (ENAA)-obtained and certified values of reference materials, mg/kg.

SRM	Sample	Ele-ment	Obtained	Certified	SRM	Sample	Ele-ment	Obtained	Certified
667	1632c	Na	324 ± 16	298.8 ± 4.8			As	15.7 ± 2.7	17.1 ± 2.8
1547	1575a	Mg	1184 ± 213	1060 ± 170	2711	667	Se	1.76 ± 0.33	1.52 ± 0.14
1633b	1547	Al	240 ± 10	249 ± 8	667	2711	Br	20.5 ± 1.0	18.7 ± 0.4
1575a	1547	Cl	411 ± 33	360 ± 19	667	1632c	Rb	147 ± 52	110 ± n/c
2711	1632c	K	835 ± 169	1100 ± 33.0	1632c	2711	Sr	53.6 ± 5.9	63.8 ± 1.4
1547	1633b	Ca	14800 ± 1420	15100 ± 604	2711	1632c	Sb	19.8 ± 1.4	19.4 ± 1.8
667	1632c	Sc	2.33 ± 0.14	2.905 ± 0.036	667	2711	Cs	0.485 ± 0.05	0.594 ± 0.01
1633b	2710	Ti	2724 ± 354	2830 ± 100	667	1632c	Ba	867 ± 95	726 ± 38
1633b	1547	V	0.38 ± 0.04	0.37 ± 0.03	1632c	667	La	35.5 ± 11	40 ± n/c
1632c	667	Cr	147 ± 13.4	178 ± 16.0	1632c	2711	Ce	9.5 ± 1.3	11.9 ± 0.2
2710	1575a	Mn	503 ± 25	488 ± 12	667	2711	Sm	0.779 ± 0.06	1.078 ± 0.028
667	2711	Fe	27220 ± 1361	28900 ± 1734	667	1632c	Eu	0.089 ± 0.036	0.1238 ± 0.0033
667	1632c	Co	3.41 ± 0.2	3.48 ± 0.2	667	1632c	Hf	11.6 ± 3.5	7.3 ± n/c
667	2711	Ni	19.6 ± 2.0	20.6 ± 1.1	1632c	2711	Th	1.32 ± 0.07	1.4 ± 0.03
2710	1572	Cu	14.8 ± 5.3	16.5 ± 1.0	667	1632c	U	0.372 ± 0.03	0.513 ± 0.012
667	2711	Zn	332.8 ± 26.5	350.4 ± 13.0					

**Table 2:** Concentration of the main elements (mg/kg//σ, %) in the wild.

	Species Site	Samples of *Tribulus terrestris* L.				Reference Plant Market, 1991 [37]
Elem		1	2	3	4	
Na		**1030**//3	**643**//3	108//3	146//3	150
Mg		**5190**//2	**5320**//2	4290//2	5410//2	2000
Al		714//4	**805**//4	112//4	41//4	80
Cl		**7350/**/8	**8540**//8	4120//8	1240//8	2000
K		**36,400**//9	**30,600**//9	26,000//9	28,500//9	19,000
Ca		**21,400**//7	**21,800**//7	**34,600**//7	19,600//7	1000
Sc		0.16//6	0.12//6	0.03//18	0.02//18	0.02
V		**1**//12	**1**//12	0.2//12	0.2//12	0.5
Mn		40//5	38//5	19//5	112//5	200
Fe		**613**//10	**482**//10	**246**//10	111//10	150
Co		0.3//10	0.27//9	0.17//11	0.17//10	0.2
Zn		**75**//5	35//5	31//5	**140**//5	50
As		0.8//3	0.7//3	0.8//3	1//3	0.1
Se		**0.302**//16	**0.203/**/20	**0.375**//15	**0.29**//16	0.02
Rb		6//17	9//17	7//17	**88**//11	50
Sr		**309**//7	**226**//7	**161**//7	51.6//7	50
Mo		**1.8**//30	0.9//30	**2.6**//30	0.2//30	0.5
Cd		0.112//20	0.064//20	0.055//20	0.051//20	0.05
Cs		0.072//7	0.117//5	0.061//8	0.034//10	0.2
Ba		33//6	30//5	27//7	3//20	40
La		0.37//7	0.39//6	0.11//10	0.05//20	0.2
Sm		0.056//12	0.058//12	0.012//13	0.007//13	0.04
Hf		0.17//20	0.12//20	0.06//20	0.009//20	0.05
Ta		0.014//20	0.008//20	0.006//20	0.009//20	0.001
Th		0.11//11	0.122//11	0.025//15	0.019//10	0.005
U		0.052//8	0.058//6	0.017//14	0.003//20	0.01

*Tribulus terrestris* L. depending on the harvesting site. Note: the number of samples is described in Section 2 of work. Exceeding values are in bold format.
